# How Inflamed Is the Horse in Training? Insights into Exercise-Induced Acute Phase Response in Endurance Horses

**DOI:** 10.3390/ijms27052328

**Published:** 2026-03-02

**Authors:** Alicja Rakowska, Anna Biazik, Magdalena Sobuś, Anna Cywińska

**Affiliations:** 1 Department of Diagnostics and Clinical Sciences, Institute of Veterinary Medicine, Nicolaus Copernicus University in Toruń, Szosa Bydgoska 13, 87-100 Toruń, Poland; a_rakowska@umk.pl (A.R.); magdalena.sobus@umk.pl (M.S.); 2 Department of Veterinary Surgery, Institute of Veterinary Medicine, Nicolaus Copernicus University in Toruń, Szosa Bydgoska 13, 87-100 Toruń, Poland; anna.biazik@umk.pl; 3 Department of Basic and Preclinical Sciences, Institute of Veterinary Medicine, Nicolaus Copernicus University in Toruń, Szosa Bydgoska 13, 87-100 Toruń, Poland

**Keywords:** exercise-induced acute phase response, cytokines, acute phase proteins, monitoring of endurance training

## Abstract

The article aimed to review the current literature analysing the complexity of an exercise-induced acute phase response in athletic horses undergoing intense training and endurance competitions. Since the endurance discipline demands physical fitness, exceptional health and excellent adaptation to an increasing workload, diagnostic methods of assessing the factors mentioned above are highly required. Athletic horses in endurance training undergo numerous metabolic but also immune adaptations, including changes in pro-inflammatory and anti-inflammatory cytokine levels. The inflammatory reaction resembles typical inflammation only to some extent; therefore, the term exercise-induced acute phase response (APR) has been introduced. Among many biomarkers, acute phase proteins (APPs), like serum amyloid A (SAA) and different types of cytokines, especially interleukin 1 (IL-1), interleukin 6 (IL-6), and tumour necrosis factor α (TNF-α), appear to play a key role. These markers may be modulated by many factors; however, proper training seems to result in the occurrence of an “anti-inflammatory state”, which is beneficial for the horse’s health and highly required in high-performance equine athletes. Further understanding of the inflammatory reaction associated with extreme physical effort is crucial for ensuring the long-term career and welfare of endurance horses.

## 1. Introduction

Endurance competitions have been recognised by the Fédération Equestre Internationale (FEI) since 1978, and in many countries, it is the most popular equestrian discipline. It is based on the controlled cross-country run on long distances, ranging from 20 to 160 km, completed in the shortest possible time according to the regulations of certain competitions. Competing at the longest distances is one of the most physically demanding activities, and as such, it requires good health, exercise capacity and proper adaptation to the increasing workload during training rather than technical skills. The horses compete in distance classes, beginning from the age of four; horses older than nine years can compete at any distance and any type of competition. Minimal age requirements are established to prevent participation of insufficiently trained youngsters. Success in this sport is determined not only by the speed but mainly by the horse’s fitness and health, which are examined by a veterinary team after each loop. Animals showing gait irregularity or signs of metabolic disorders are eliminated from further competition as they failed to qualify. Any breed of horse can participate, but pure-bred Arabians generally dominate, especially at the top levels, due to the breed’s stamina and natural endurance abilities.

## 2. Effort Character in Endurance Training

Horses are introduced to endurance training no earlier than age four, and often have long sport careers, achieving the best results at the ages of 8–16 years. Horses prepared for long-distance rides (120–160 km) undergo several years of training, throughout which they compete in lower rank events on shorter distances. Throughout this period, metabolic changes take place, allowing adaptation to an increasing workload. Exertion in endurance rides is long-lasting (significantly more than an hour) and of an aerobic nature. Thus, the energy for muscle work comes mainly from free fatty acids, which are utilised in processes involving oxygen use, resulting in significant heat loads and sweat losses. The workload applied during training and elite endurance competitions results in numerous changes in the horse’s metabolism, initiating the phenomenon known as the exercise-induced acute phase response (APR). It is similar to the acute phase response observed in inflammation; however, substantial differences have been noted, and its purpose, advantage or impairment to the horse is not fully understood.

## 3. Common Health Checks of an Endurance Horse

Health and performance ability of the horse are usually assessed based on physical examination, monitoring of heart rate at rest and measurement of selected haematological parameters. In endurance horses, primarily creatine kinase activity (CK), packed cell volume (PCV) and concentrations of total protein, glucose, phosphorus and potassium [1,2,3] are measured. The analysis of these parameters enables assessing the general health and metabolic changes resulting from training progress; however, it does not provide the necessary information for identifying subclinical disorders. These may worsen with effort and result in disruption in training or even termination of the horse’s sport career. Thus, measuring other markers that reflect more aspects of the horse’s health and condition is being investigated [3,4,5,6,7,8,9,10,11,12]. The most common cause of training interruption and elimination from competition is lameness, typically resulting from the overload of the musculoskeletal system [3,13,14]. Novel markers, such as serum amyloid A (SAA), the main acute phase protein (APP) in horses, have been proposed as an indicator of subclinical disorders that may result in lameness during long-distance endurance rides [10,12,15]. In case of overload, which is usually a consequence of inappropriate training or overtraining, clinical symptoms (visible lameness) may be preceded only by a slight decrease in the horse’s sport condition and performance, which can remain unnoticed by a trainer. The effects of microinjuries may accumulate, which leads to shortening of the competing period and results in clinical pathologies that require long-term treatment. It has been proposed that the high-intensity training, which induces microinjuries in muscles, tendons or joints, also elicits a systemic inflammatory response, displayed by exercise-induced acute phase response [9,10] and the changes in neutrophil and lymphocyte activities. This reaction, described after prolonged strenuous exercise in human, canine and equine athletes [10,15,16,17,18,19,20,21], is similar, but not identical to the typical acute phase response in inflammation.

## 4. The Mechanism of the Acute Phase Response

Acute phase response (APR) is defined as the first, rapid and nonspecific response to any disturbances in homeostasis, promoted by pro-inflammatory cytokines released at the site of injury, and vascular changes which activate inflammatory cells [22,23,24,25,26]. These responses result in the enhanced production of cytokines and inflammatory mediators that diffuse into the extracellular fluid and circulate in the blood. Pro-inflammatory cytokines, mainly interleukins (ILs) such as IL-1, IL-6, IL-8 and tumour necrosis factor α (TNF-α), stimulate the systemic responses leading to the activation of defence and adaptation mechanisms focused on eliminating invasive agents and the repair of damaged tissues. These responses include neuroendocrine, behavioural and metabolic changes and manifest as clinical signs like fever, anorexia or muscle catabolism, and laboratory signs such as neutrophilic leucocytosis and changes in blood protein concentrations [22,23,24,25,26,27]. Pro-inflammatory cytokines stimulate APPs synthesis in the liver within a few hours of initial stimulation [22,23,24,26,28,29]. Further changes in the APPs concentrations in blood reflect the state of the inflammatory process [22,26,28] and allow for the evaluation of the health status, severity of the disease, monitoring of the recovery period and detection of subclinical diseases. The biological functions of APPs are not fully understood, although they appear to play a major role in the modulation of the inflammatory process and promotion of tissue healing. The shift towards the production of different APPs is promoted by at least five redundant cytokines [29], but TNF-α, IL-1 and especially IL-6 play a key role in this process by activating specific hepatocytic receptors [22,23,29]. In horses, blood concentrations of fibrinogen, C-reactive protein (CRP), haptoglobin and especially serum amyloid A (SAA) are considered the most important [13,16,22,23,24,26,30,31,32,33,34]. IL-1 type cytokines (including IL-1 and TNFα) elicit a primary auto-stimulatory signal, which induces the synthesis of first-line, rapid-reacting APPs, including CRP and SAA. The secretion of IL-6, acting as a secondary signal, promotes the synthesis of the second-line APPs, such as haptoglobin and synergistically induces the production of type 1 APPs, but also reduces the production of IL-1 type cytokines. In contrast, type 2 APPs are neither induced nor synergistically stimulated by IL-1 [22]. The reaction is limited spontaneously by IL-1, IL-4, some of the APPs and locally by Kupffer cells, which secrete either IL-6 (when stimulated by pro-inflammatory cytokines) or IL-10 (causing suppression of the local IL-6 production) [23].

## 5. Exercise-Induced Acute-Phase Response

In humans, dogs and horses, the reaction analogous to the acute phase response has been described after prolonged strenuous exercise [10,12,15,16,17,18,19,21,35,36]. Exercise-induced APR seems to be species-specific and differs from typical acute inflammation, as the concentrations of only several blood parameters increase. APR also varies depending on the type, intensity and duration of the exercise [10,15,16,18,19,37,38,39]. In humans, exercise-induced APR involves changes in the concentrations of cytokines like IL-6, APPs like CRP, fibrinogen, haptoglobin or hepcidin and CK [16,17,36,40]. In horses, exercise-induced APR has been reported mainly as a significant change in SAA concentration (present at trace levels in healthy animals) and an increase in pro-inflammatory cytokines in blood [13,22,30,31,32,33,34]. Several theories explaining the primary cause of exercise-induced APR and exercise-induced inflammation have been proposed; however, all of them were based on human physiology. One of the mechanisms proposed for exercise-induced inflammation involves high-volume/intensity training, which is responsible for muscle, skeletal and/or joint trauma, activating circulating monocytes and other cells to produce pro-inflammatory cytokines such as TNF-α, IL-1, and IL-6 [41,42]. These peripherally originated cytokines can, in turn, increase or exacerbate localised inflammatory responses within the musculoskeletal system [17]. Interestingly, the enhanced expression of TNF-α was confirmed to occur in racehorses after intense bouts of exercise, but not frequently during endurance training; on the contrary, even a decrease in TNF-α was described [43,44]. However, increased TNF-α values were observed in more experienced horses, as well as slower finishers [45]. Alterations in the expression of IL-6 were demonstrated in racehorses of different abilities, but were not directly confirmed in high-level endurance horses, although higher IL-6 concentrations might be specifically linked with the higher speed of the eliminated horses [43]. Previously, increased IL-6 concentrations were also connected with more experienced horses [45]. Other authors [46] presented the theory of “danger exercise”, when activated leukocytes become overreactive to exercise-based stimulation. It has been suggested that systemic acidosis, heat production, and reduced pO_2_ during exercise can stimulate hypoxia-inducible factor and dysregulate neutrophil functions, causing them to release potentially damaging cytokines and exacerbate, rather than ameliorate, chronic inflammation [46,47]. Additionally, strenuous exercise has been demonstrated to induce detectable endotoxemia in both humans and horses, and a following toll-like receptor 4 (TLR4) expression peak [43,45]. It can be attributed to either Gram-negative bacteria multiplication or weakening of the intestinal mucosal barrier; however, both mechanisms seem positively correlated with increased plasma acid concentration, decreased intestinal blood perfusion and gastrointestinal symptoms, which are also usually more prominent in the less successful human athletes [45]. Triggering an inflammatory reaction affects the skeletal muscle status, including muscle mass, which is strictly related to athletic potential. Pro-inflammatory cytokines act in two different ways: they induce muscle atrophy, but also promote regeneration and increase muscle mass [48,49,50,51]. Muscle damage is confirmed by an increase in creatine kinase (CK) activity, which is well known to occur after prolonged exercise and its significantly elevated concentration is supposed to be negatively correlated with the probability of completing the endurance competition [52]. Although it is not sufficient to evaluate the extent of muscle damage, it suggests the presence of membrane leakage in myocytes [16]. The type and severity of muscle damage that may trigger the inflammatory response are difficult to define precisely; however, even necrosis of muscle fibres after a marathon run in humans has been reported [53]. While primarily pro-inflammatory cytokines like IL-1 and TNF are always present during exercise, their levels elevate significantly in excessive exercise, which was described as a ‘Cytokine Hypothesis of Overtraining’ [42,54,55]. Other authors suggested that the inhibition of satellite cells’ functions by IL-6 and TNF-α in cases of prolonged exposure may reduce muscle growth in young horses [51]. It was also proposed that the response of satellite cells is an important mechanism of muscle adaptation to exercise. Currently, it is favoured that properly regulated intramuscular inflammatory responses are integral for muscle repair and regeneration [56]. The role of various inflammatory cells that control myogenesis and extracellular matrix remodelling is taken into consideration [56,57]. It might be possible that the appropriate balance between exercise and rest is reflected in the body by the expression of anti-inflammatory cytokines, allowing for adaptation to increased workload. Two signal transduction pathways, the mitogen-activated protein kinases (MAPK) and nuclear factor-κB (NF-κB), upregulated by muscle contraction, have been suggested to be involved in these reactions [47,58]. NF-κB, influenced by IL-10, reduces the release of pro-inflammatory cytokines, including TNF-α and IL-1β [59]. Also, an increasing number of authors claim that adipose tissue producing adipokines [60] and skeletal muscles producing myokines [61] can be recognised as endocrine organs regulating metabolism and secreting interleukins IL-6, IL-8 and IL-15 [61]. It is also widely acknowledged that exercise-induced APR results from glycogen depletion in working muscles and skeletal muscle damage [16]. In response to this energy crisis, IL-6 acts as both a myokine and an adipokine, recognises low muscle glycogen during exercise and increases hepatic glucose production by lipolysis [61,62]. In humans, IL-6 and IL-15 are specifically described to have a divergent pro- or anti-inflammatory impact on muscle tissue, controlled by up-regulation of certain receptors [54]. IL-15 is recognised as a growth factor with anabolic effects on increased myosin production in muscle tissue [63,64]. Theories described above might also be valid for the horses; however, substantial differences in the metabolism and species-specific pattern of acute phase response have to be taken into consideration and studied in the near future. The most commonly described markers of inflammation in horses are collected in Table 1. The majority of them, but also many others mentioned above, like IL-15 or NF-κB, appear to be crucial in equine exercise-induced APR, but still most of our current knowledge is solely based on hypotheses extrapolated from human research.

## 6. Acute Phase Proteins in Endurance Horses

The 10-fold or higher increase in SAA, the most clearly confirming APR [22,23,24,28,30,31,34,67], has been reported in endurance horses that completed the longest (120–160 km) distances [6,10,12,38]. The unique feature of SAA concentrations was the proportional increase in relation to covered distance [38], whereas moderate equine APPs, haptoglobin and CRP remained unchanged [10]. Major APPs are more sensitive to inflammatory stimuli, whereas moderate APPs do not elevate as dramatically [26]; however, the proteomic studies conducted by other authors in horses after long-distance endurance rides revealed changes in the concentrations of minor acute phase proteins, including α-1 antitrypsin, ceruloplasmin and α-2 macroglobulin and other plasma proteins, involved in pathways related to inflammation, coagulation, immune modulation, oxidant/antioxidant activity and cellular and vascular damage [8]. Additionally, it confirms the systemic character of exercise-induced APR, although displayed to a lesser extent than typical APR and essentially different from the one reported in humans and dogs. Measurement of SAA concentrations may be useful to detect pathology that has not yet resulted in clinical symptoms; however, subclinical issues might exacerbate during the long, exhausting ride and result in elimination at the vet gate during the competition. It has been reported that horses with pre-competition SAA concentrations higher than 1 mg/L failed to complete their long-distance rides [12]; however, other authors did not observe such a phenomenon [5]. Thus, measuring the serum SAA concentration before entering a competition may indicate whether the horse is in poor condition; however, the exact time when the SAA level should be determined before competitions remains unclear. High SAA concentrations in the finishers of the longest distances seem to be a justified response to this kind of extreme effort. However, triggering such an inflammatory response during the competitions usually does not result in serious clinical disturbances, so the routine management of the horse after competition should ensure proper health care and welfare. Endurance rides on shorter distances (up to 60 km), as well as intensive endurance training, produced no changes in CRP and haptoglobin concentrations and an insignificant increase (up to 2-fold) in SAA concentrations in experienced horses, prepared to compete at the longest distances [10,15]. According to the authors, this cannot be interpreted as the exercise-induced APR. The training was designed to obtain the exertion similar to that of moderate distance competitions, which has been previously reported not to produce any changes in APPs concentrations [10]. In contrast, intense training at 50 km distance produced an increase in SAA concentration by 4-fold in young horses at the beginning of their endurance careers, and this change was considered relevant and confirmatory for systemic reaction; however, not high enough to clearly indicate pathology [15]. On the other hand, the reaction stimulated in such conditions may be a part of physiological adaptation to increased workload during training. The interpretation of this fact based on current knowledge is difficult, as the data dealing with the impact of regular training on the onset of exercise-induced APR is limited and conflicting results have been presented. It should also be taken into consideration that measurements of APPs in blood reflect the systemic response resulting from muscle and tendon microinjuries, but do not give any insight into local processes in the joints, ligaments and bones [68]. Thus, it is not clear if exercise-induced APR after intense training and competitions might contribute to local chronic processes while the horses remain clinically healthy and fit to compete.

## 7. Pro-Inflammatory Cytokines in Endurance Horses

Exercise-induced APR in endurance horses has also been confirmed by the changes in cytokine levels, which provided additional information about its mechanism [6,7,38,66]. The increases in IL-6 and IL-10, but not IL-1 and TNFα, have been previously reported after long (120–160 km) distance endurance competitions, suggesting that the response was promoted by type 1 cytokines. It was also suggested that exercise-induced APR in the horses in regular training is accompanied by a strong anti-inflammatory response, which is in line with the hypothesis of “anti-inflammatory state” [9,38,66,69,70]. It cannot be excluded that the lack of changes in IL-1 and TNF-α was caused by an inappropriate sampling time. Interleukins act as a cascade, so that soon after the given stimulus, TNF-α and IL-1β might have been increased and stimulated the liver to produce acute phase proteins [38]. Then secondary stimulation by IL-6 promoted SAA synthesis and then suppressed IL-1 and TNFα production, which was detectable at the sampling time after long-distance competitions [66]. It seems that horses undergoing repeated bouts of exercise develop strong adaptation mechanisms, capable of maintaining an anti-inflammatory body condition at rest [71]. Such a state was confirmed to develop after the 3rd month in a recent longitudinal study of young horses undergoing initial endurance training, depicting adaptation to an increased workload [44]. This beneficial effect may be disrupted by intense effort; however, it should be overcome in less than 24 h in well-trained individuals, suggesting that specific training may increase adaptive response to exercise [72,73]. Long-term influence on commonly described cytokines is presented in Table 2.

## 8. Modulation of Inflammatory Gene Expression in Endurance Horses

Changes in the pathways involved in inflammatory and immune responses have been confirmed in sport horses by microarray studies [4,11,74], with the modulatory effect of exertion proven at the genetic level [11,74,75,76]. In endurance horses, strong modulation in expression of 132 genes (97 upregulated and 35 downregulated) has been identified within 24 h of 90–120 km endurance competitions. The main pathways and functions affected were related to immunological and inflammatory responses, with a relative abundance of cytokines modulating the activities of the cells involved in inflammation [73]. The same authors revealed that *IL1R2*, *MMP1* and *MMP13* genes achieved high expression values immediately after and 24 h after national and international endurance competitions (90–120 km). The maximum down-regulation 24 h after exercise was found for *COX7A1*, *COX7A2L*, *CCL6*, *TRAK1*, *HIP1* and *SLC25A10* [74]. Gene expression in peripheral leukocytes in endurance horses has been shown to correlate with performance and common disorders. One study revealed that after 140–160 km endurance rides, 62 genes were differentially expressed between the horses that completed the distance and those that were eliminated at the vet gates [4]. More genes were up-regulated in horses that finished the rides, while down-regulated genes predominated in eliminated horses. In the latter group, the expression levels of 28 and 50 genes were significantly correlated with CK and Aspartate-Aminotransferase (AST) activity, respectively. These genes were expressed in relation to the clinical onset of rhabdomyolysis and haemolysis [4]. Interestingly, another study described more enhanced upregulation in genes responsible for the production of inflammatory proteins in geldings compared to mares during long-distance competition (160 km), including IL-8 and TLR4, ligand for endotoxemia-inducing lipopolysaccharides. In the meantime, mares were discovered to upregulate mostly genes responsible for protein synthesis and muscle repair [77]. Results that confirm the role of inflammatory cytokines have also been reported in horses undergoing shorter exertion [9,78]. Two different patterns of cytokine profiles have been described in the blood and muscles of unconditioned horses after treadmill exercise [78]. In blood, the transient increases in mRNA expression for TNF-α and IL-1 have been observed immediately after exertion and 2 h later, respectively. In muscles, an increase in mRNA for TNF-α and IL-6, but not IL-1, occurred. The cytokine pattern in blood matches type 1 APR. The different pattern in muscles was consistent with the findings presented in human and equine studies [79,80,81], and it has been postulated that upregulation of IL-6 was of metabolic origin and did not reflect APR resulting from muscle damage [78]. On the other hand, the upregulation of mRNA for IL-1 and IL-6 has been detected in the blood of the young Thoroughbred horses following exercise bouts [9]. The increased expression of IL-1β was dependent on exercise intensity and related to the damage of muscle fibres, as indicated by the concentration of malondialdehyde. The expression of IL-6 mRNA was inversely correlated with mRNA of pro-inflammatory cytokines, suggesting the mechanism that generated an anti-inflammatory environment [9]. Moreover, the baseline expression of type 1 cytokines (IL-1β and TNF-α) decreased with training, which confirmed the overall “anti-inflammatory state” hypothesis [44,69,70,71,82]. In endurance horses, upregulation of the genes for matrix metalloproteinase-1 (*MMP1*) and IL-8 (*CXCL8*), derived from previous genome-wide expression analysis, was found after 90–120 km rides [11]. It has been suggested that the genes expressed at higher levels were more likely to respond to the homeostatic changes induced by the ride, and further production of mRNA transcripts was unnecessary. Thus, it can be interpreted as an adaptive reaction [83]. Other authors [84] determined that the expression level of inflammation-related genes, including ones responsible for interleukin-1 receptor type II (*IL1R2*), matrix metallopeptidase 8 (*MMP8*), protein S100-A8 (*S100A8*), and SAA production, increased at 4 h after the race, whereas that of protein c-Fos (*FOS*) increased immediately after exercise. Unfortunately, horses were examined only before and after one race, so the onset of changes during training remained unknown. In the other study [9], young 2-year-old race horses were examined four times during training season, and time- and intensity-dependent changes in selected pro-inflammatory cytokine (IL-1, IL-6, TNF-α and IFNγ) gene expression have been observed. The general conclusions included the occurrence of adaptation to exercise, with a training period indicated by an overall reduction in the expression of pro-inflammatory cytokines; however, the expression of IL-6, which plays a key role in APR, was increased.

## 9. The Impact of Exercise and Exercise-Induced APR on the Horse’s Health

Identification of exercise-induced changes in the pathways involved in inflammatory and immune responses clearly confirmed the immunomodulatory effect of exercise. It has also been suggested as a novel mechanism by which physical activity modulates health, growth and disease risk [4,11,73,85]. The essential question is whether exercise-induced APR is a part of training progress beneficial for horse health. It is widely accepted that strenuous, high-intensity or long-lasting exercise promotes transient immunosuppression, which can lead to increased susceptibility to common infections, particularly of the respiratory system [86,87,88,89,90]. This condition has been interpreted as a result of the alterations in the number and function of circulating leukocytes; however, high individual variations in these reactions have been reported [87,91,92,93]. Several articles confirmed the suppression of both innate and acquired immunity in performance horses competing in long-distance endurance rides [10,20,88,93]. On the other hand, immunosuppression, measured by lymphocyte proliferation, has been shown in horses with decreased athletic performance, associated with slight respiratory disorders [94]. T-cell suppression in ponies subjected to 5 days of strenuous exercise programme also resulted in the reduced effectiveness of vaccination against influenza [95]. However, the administration of the vaccine to the ponies with exercise-induced immunosuppression was still safe and provided clinical protection [96]. The innate response seems to be affected by the training process rather than acute exercise and differs between the respiratory compartment and blood [97]. The authors suggested that the increased susceptibility to respiratory infections may result from a local decrease in the activity of pulmonary alveolar macrophages, measured by the decreased production of TNF-α and IFNβ. However, systemic reaction of blood monocytes was increased in trained Standardbreds, regardless of the intensity of training [97]. According to a recent study, haematologic parameters of well-prepared horses, completing 80, 120 and 160 km endurance competitions, returned to adaptive threshold within 14 days. Moreover, the article presents various ratios and indices, used in human medicine to describe inflammatory markers or exercise-induced stress and adaptive response, indicating that parameters sensitive to acute inflammation may play a key role in assessing the fitness and proper recovery of equine athletes [98].

## 10. Conclusions

In conclusion, the modulating effects of exercise-induced acute phase response are complex and depend on many, sometimes unpredictable factors. Various effects of this reaction have been reported; however, its precise role is not yet fully understood. Among many papers, the SAA, IL-1, IL-6 and TNF-α appear to be the most significant biomarkers for further concerns, regarding scientific (better understanding of the mechanisms) and maybe in future also practical (use of biomarkers for the monitoring of endurance horses) issues. The concentrations of the biomarkers varied between the studies mentioned in our review; however, a general decrease in the main inflammatory cytokines after prolonged endurance training might validate the “anti-inflammatory state” theory, whereas a certain SAA threshold defined by future studies may indicate a well-prepared, healthy equine athlete. It seems crucial to investigate—not if—but how and when the triggering of acute inflammatory response by training and/or competing is beneficial or harmful for the horse. The authors hope that the outcome of forthcoming studies on the topic will provide the answers and improvements in understanding of the health of sport horses, equine exercise adaptation and finally their welfare status.

## Figures and Tables

**Table 1:** The influence of the single endurance training session on inflammatory markers and APPs, according to the reference authors.

Inflammatory Markers and APPs	Influence of the Intense Endurance Session	Reference
creatinin kinase	increase	[4]
serum amyloid A	increase	[10]
C-reactive protein	no changes	[10]
haptoglobin	no changes/ decrease	[10]/ [65]
ceruloplasmin	decrease	[65]
calprotectin	increase	[65]
interleukin-1	increase	[66]
interleukin-6	increase	[45,66]
interleukin-8	increase	[43]
interleukin-10	increase	[38,66]
Tumour Necrosis Factor-α	decrease/ increase	[45,66]/ [38]

**Table 2:** The influence of an endurance training programme on inflammatory markers, according to the reference authors.

Inflammatory Markers	Influence of an Endurance Training Programme	Reference
IL-1	decrease	[44]
IL-6	decrease	[44]
IL-10	increase/ decrease	[38]/ [44]
IL-17	decrease	[44]
TNF-α	increase/ decrease	[45]/ [44]

## Data Availability

No new data were created or analyzed in this study. Data sharing is not applicable to this article.
